# Achieving Adaptive Visual Multi-Object Tracking with Unscented Kalman Filter

**DOI:** 10.3390/s22239106

**Published:** 2022-11-23

**Authors:** Guowei Zhang, Jiyao Yin, Peng Deng, Yanlong Sun, Lin Zhou, Kuiyuan Zhang

**Affiliations:** 1 School of Safety Engineering, China University of Mining and Technology, Xuzhou 221116, China; 2 Shenzhen Urban Public Safety and Technology Institute, Shenzhen 518046, China; 3 Key Laboratory of Urban Safety Risk Monitoring and Early Warning, Ministry of Emergency Management, Shenzhen 518046, China; 4 School of Computer Science and Technology, China University of Mining and Technology, Xuzhou 221116, China

**Keywords:** multi-object tracking, YOLOv5 object detection, improved DeepSORT, unscented Kalman filter, adaptive algorithm

## Abstract

As an essential part of intelligent monitoring, behavior recognition, automatic driving, and others, the challenge of multi-object tracking is still to ensure tracking accuracy and robustness, especially in complex occlusion environments. Aiming at the issues of the occlusion, background noise, and motion state violent change for multi-object in a complex scene, an improved DeepSORT algorithm based on YOLOv5 is proposed for multi-object tracking to enhance the speed and accuracy of tracking. Firstly, a general object motion model is devised, which is similar to the variable acceleration motion model, and a multi-object tracking framework with the general motion model is established. Then, the latest YOLOv5 algorithm, which has satisfactory detection accuracy, is utilized to obtain the object information as the input of multi-object tracking. An unscented Kalman filter (UKF) is proposed to estimate the motion state of multi-object to solve nonlinear errors. In addition, the adaptive factor is introduced to evaluate observation noise and detect abnormal observations so as to adaptively adjust the innovation covariance matrix. Finally, an improved DeepSORT algorithm for multi-object tracking is formed to promote robustness and accuracy. Extensive experiments are carried out on the MOT16 data set, and we compare the proposed algorithm with the DeepSORT algorithm. The results indicate that the speed and precision of the improved DeepSORT are increased by 4.75% and 2.30%, respectively. Especially in the MOT16 of the dynamic camera, the improved DeepSORT shows better performance.

## 1. Introduction

Nowadays, vision-based object tracking has a wide utilization of applications in behavior recognition, autonomous driving, and intelligent monitoring [1]. With the influence of background, illumination, attitude changes, fast motion, and partial occlusion, accurate and robust object tracking has great significance. Although the existing visual object tracking has made significant progress, for multi-object tracking (MOT) in complex scenes, there are often challenges such as mutual occlusion, background interference, and drastic changes in motion states. The multi-object tracking is still a hot and challenging research [2].

Many excellent methods have been proposed for object tracking. Although these methods are effective and improve tracking accuracy, they suffer from one or more of the following limitations. In general scenarios, correlation filters and their improvements [3,4,5] present satisfactory performance in tracking a single object. For multi-object tracking, each object requires to be allocated a tracker, which consumes extensive CPU resources. In addition, the object tracking methods based on deep learning have also attracted much attention. For example, Fast RCNN [6], Faster RCNN [7], MDNet [8], Mask RCNN [9], Siammot [10] and other algorithms are used for object tracking. Although they have achieved high precision in multi-object tracking, it consumes more computing power and cannot fully guarantee real-time performance.

With the improvement of the detection algorithm YOLO [11] to the latest YOLOv5, detection-based object tracking frameworks, such as Sort [12], DeepSORT [13], fully meet real-time performance while maintaining accuracy. Since the performance of tracking often depends on object detection. Therefore, they focus on improving object detection performance in previous studies. By improving YOLOv4 and combining the DeepSORT algorithm, the accuracy of vehicle tracking is improved [14]. In [15], a multi-node tracking (MNT) framework suitable for most trackers is proposed, and a cyclic tracking unit (RTU) is designed to score the potential trajectory through long-term information. In addition, a motion feature-based SORT algorithm (MF-SORT) is [16] proposed, which focuses on the characteristics of moving objects during information association and maintains a balance between efficiency and performance.

Although some studies have improved the DeepSORT algorithm, as shown in [17], which is proposed to combine low confidence trajectory filtering and depth correlation measurement (depth ranking) algorithm into simple online real-time tracking. However, the motion trajectory cannot be correctly predicted and updated by the classical Kalman filter in the DeepSORT. Due to the interference of occlusion, noise, and background factors, there is almost no linear motion for the objects. The nonlinear error is inevitable in multi-object tracking, and the classical Kalman filter ignores these errors, which reduces the robustness of multi-object tracking. In addition, the detection algorithm directly affects the performance of the tracking algorithm, and these factors will also lead to a sharp decline in object detection accuracy. The classical Kalman filter does not have the ability to distinguish and correct the outliers of the detection algorithm, resulting in the poor robustness of the DeepSORT algorithm based on the classical Kalman filter.

This paper aims to propose an improved DeepSORT tracking algorithm to achieve high accuracy and robust multi-object tracking. The latest YOLOv5 with high accuracy is utilized as the object detection algorithm to extract feature information, and a generic object tracking model is designed based on the object motion state first. Then, the unscented Kalman filter (UKF) based on the generic tracking model is designed to predict and update multiple objects, which reduces the nonlinear errors. In addition, we devise an adaptive outlier detection algorithm to adjust the observation noise covariance matrix, which improves the robustness of the DeepSORT object tracking algorithm. Specifically, we summarize the contributions of this paper as follows.

- Through the in-depth study of image motion characteristics, a general accelerated motion model for multi-object is provided, which is similar to the variable acceleration motion. In addition, a multi-object tracking system based on the unscented Kalman filter is established to enhance tracking accuracy.
- Aiming at the occlusion in the tracking process, an improved DeepSORT algorithm with the adaptive factor is designed to improve the tracking robustness. The algorithm can adapt to the fast motion of objects better and reduce the observation noise caused by occlusion.
- We conduct extensive experiments to indicate the tracking performance. The improved DeepSORT algorithm is compared with DeepSORT on the MOT16 data set. In addition, the results indicate that the proposed improved DeepSORT has better tracking speed and accuracy, especially with the dynamic cameras.

The rest of the paper is arranged as follows. In Section 2, we introduce the related work. Section 3 describes the detection-based object tracking methods. The following is the general object tracking model in Section 4. Section 5 presents the improved DeepSORT algorithm with the unscented Kalman filter. Section 6 reports the experiments and evaluation. We finally summarized our work in Section 7.

## 2. Related Work

### 2.1. The Object Detection Methods

The convolutional neural network (CNN) achieves incredible success in object detection and has a strong ability to capture visual features. The CNN-based object detection methods can be divided into two classifications: (1) the methods consist of two stages. First, the candidate frames containing objects are identified in the first stage, and the object classification is carried out by using the CNN network in the next stage. (2) The one-stage method is directly transformed into a regression problem to determine the position of the object. Some typical two-stage issues include SPP-Net [18] based on an appropriate spatial pyramid idea, allowing CNN to have different input sizes and a fixed output. Nas-FPN [19] improves the extraction method and feature selection. A single shot multi-box detector (SSD) [20], and you only look once (YOLO) are the most famous single-stage methods.

In addition, YOLOv2 [21] adds a batch normalization layer to speed up network convergence. YOLOv2 also eliminates the complete connection layer, allowing the training of input images of any size. It is a method of enhancing data to train models. YOLOv3 [22] on the basis of YOLOv2, adopts multi-label classification and uses logistic regression instead of the softmax function to calculate the image input belonging to the specific categories. Using binary cross entropy loss value helps to reduce computational complexity. YOLOv4 network [23] integrates many new modules to make training and target detection effective and powerful, including weighted residual connection, cross-phase partial connection, enhancing the new hub of CNN learning, cross batch normalization, improved version of CBN, self-confrontation training, so as to obtain better accuracy.

CNN structure shows excellent performance in detection tasks, but CNN is very susceptible to the scale variety of objects [24,25]. Compared with the two-stage scheme, the one-stage uses a grid for object prediction, and the limitation of grid space reduces the prediction accuracy, especially for small objects.

### 2.2. The Object Tracking Algorithm

Detection-free tracking (DFT) and detection-based tracking (DBT) are the most utilized for tracking to initialize objects. Before the tracking, the DBT method with background mode detects the moving objects in frames. The DFT requires initializing the tracking, but it cannot deal with deleting old objects and adding new objects. Due to the progress in object detection, the detection-based tracking method has become the main tool to track multi-object video data quickly and accurately. In this model, when a series of videos are processed at the same time, the object trajectory is usually determined by the global optimization problem. Previous schemes such as joint probabilistic data association filter (JPDAF) [26] and multiple hypothesis tracking (MHT) [27] link data on a frame-by-frame basis. Two recent methods [28,29] improve the tracking based on discovery and show good results. However, the performance of these algorithms has a high computational cost and complex implementation.

Generally speaking, the object tracking algorithm consists of two sections, including the object detection algorithm, which gives the detection results in each frame and is based on the information association algorithm to decide if the detection is associated with the existing state estimation. A low confidence tracking filter is proposed to be combined into the real-time and simple tracking of DeepSORT [17]. In addition, self-generate a data set for data association is utilized for training the convolutional neural network. An IOU tracker is proposed in [30], which uses a greedy algorithm to correlate the detection from subsequent frames whose cross union (IOU) is greater than the threshold into trajectories. By adding visual single-tracking, the IOU tracker is extended to the V-IOU tracker, which helps the IOU tracker solve the lost detection and reduce ID switch numbers and fragments. The tracker initializes when there is no detection associated with the current frame and stops when the new detection meets the IOU threshold [31]. Ref. [32] proposes a deep learning framework that is utilized to automatically perform the task of monitoring social distance using surveillance video. The framework uses the YOLOv3 model to separate persons from the background and uses the DeepSORT method to track the identified objects with the help of the bounding box and the assigned ID.

The implementation of the DeepSORT algorithm, which combines the detection framework composed of YOLOv3 and RetinaNet on the VISDRONE 2018, uses the camera installed by UAV to capture mot in various scenes [33]. The quality of the detection methods is essential to the multi-object tracking model. The dependence of the object tracker on the accurate detection model is proposed in [34]. Dividing tracking into detection/prediction and data management between frames can alleviate the degradation of real-time object-tracking performance. Therefore, ref. [35] propose a pre-trained support vector machine (SVM) and an optical flow-like equation to detect objects and the correlation between frames. A Bayesian filtering framework based on a change point detection method is proposed in [36]. The KLT-based detector is used to calculate the foreground area as the occlusion detection. A real-time 3D object attitude tracking algorithm is devised, which utilizes the Gauss-Newton method to optimize the region-based loss function [37].

The tree structure is used to model and propagate multiple CNN to determine the object state to update the path in consecutive frames, but this becomes more complex for mobile cameras [38]. Therefore, ref. [39] proposed a real-time tracking scheme in a highly dynamic environment for self-service robot control. A relative motion network (RMN) is constructed by the relative motion between objects to eliminate the influence of accidental camera motion [40]. A hierarchical data association, including spatial information and appearance information, is proposed, which has been successfully applied to the detection and tracking of candidate selection [41]. A multi-step data association is proposed in [42], which includes spatial distance and short-term local association, global data association with appearance model, and occlusion processing with trajectory.

### 2.3. YOLO and DeepSORT Applications

Detection-based tracking methods have been widely utilized in academia and industry. At present, most object tracking schemes take the image edge features and probability density as tracking standards. Therefore, the object search direction is along the rising direction of probability gradient, such as particle filter [43]. However, these algorithms cannot work in complex environments. An object tracking algorithm in sports-related fields based on YOLOv4 and DeepSORT is proposed to establish a tracking framework for players in the game and find deficiencies [44]. Based on the classical detection and tracking algorithm, a dynamic pedestrian tracking scheme utilizing YOLOv5 and DeepSORT is devised to improve the tracking accuracy and realize the real-time monitoring of pedestrians in video [45]. Similarly, ref. [46] also proposed a personnel tracking framework using DeepSORT.

Unlike object detection frameworks such as CNN, it can not only timely detect but also monitor the tracks of objects according to the learned information until they leave the camera. A DeepSORT algorithm with YOLOv5-based is also proposed in [47]. When using the Hungarian scheme to match the same object, the Kalman filter is used to predict positions. Using an RGB camera to build the sight distance system of the transplanter machine, YOLOv3 and DeepSORT are utilized to detect and track obstacles and find out the center position of paddy field obstacles [48]. Ref. [49] introduced the use of improved YOLOv3 and DeepSORT tracking algorithms to detect and track ships. K-means clustering method and soft non-maximum suppression are introduced to optimize the initial value of the anchor box and deal with the insufficient screening of candidate frames. The variants of the detection model YOLOv4 and the tracking algorithm DeepSORT, a powerful pear counter in real-time, are generated for mobile applications [50]. An adaptive model combining YOLOv4 and DeepSORT is developed [51]. It makes use of the advantages of tracking and pays attention to the simplicity and effectiveness of the algorithm, high accuracy of object detection, and fast calculation time.

## 3. Existing Detection-Based Multi-Object Tracking Method

DeepSORT is a common multi-object tracking algorithm with detection-based. In this paper, YOLOv5 [52] is utilized as the object detector, and its output is used as the observation to update the Kalman filter in DeepSORT. This section first introduces the network structure of YOLOv5. Then, we briefly describe the algorithm framework of DeepSORT, and finally, we give a classical object tracking model.

### 3.1. The Object Detection of YOLOv5

**Figure 1:**
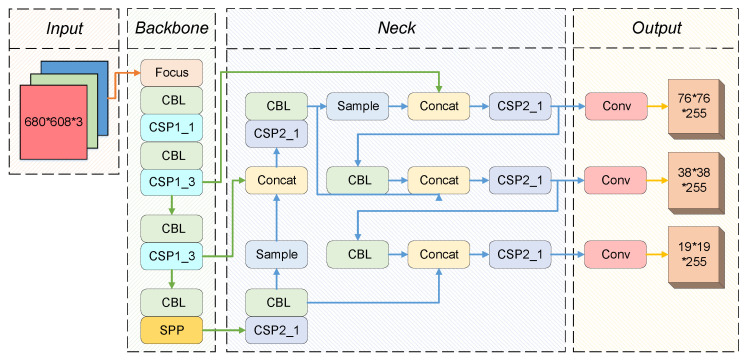
The structure of YOLOv5.

The network structure of YOLOv5 is presented in Figure 1. We can see that the YOLOv5 consists of the backbone network, neck network, and head output, which are utilized for feature extraction and fusion, object detection, respectively. The Backbone layer extracts feature mappings of different sizes from the input images by multiple convolutions and pooling. The Neck network utilizes the pyramid structure of FPN and PAN to fuse features at different levels, which enhances the capability of feature fusion. From these new feature mappings, The Head networks perform object detection and classification. The CBL module in YOLOv5 mainly consists of convolution, normalization, Leaky activation function, etc. Two cross-stage partial (CSP) improve inference speed and accuracy by reducing model size. In addition, the Spatial Pyramid Pooling module (SPP) performs maximum pooling and concatenates features for fusion.

### 3.2. DeepSORT Object Tracking Algorithm

Similarly, we introduce the DeepSORT object tracking algorithm, which consists of three parts, prediction, observation, and update, respectively. Firstly, we predict the bounding box of the object in the current frame by using the Kalman filter. Meanwhile, we detect the frame through YOLOv5 if the predicted bounding box is determined. Then, we correlate the data between the detection result and the prediction. We update the tracked bounding box utilizing the classic Kalman filter after successful matching. Finally, the object box of the next frame is predicted according to the current frame, and the cycle continues. If the predicted box fails to match with the detection result, the prediction and the detection bounding box that failed to match are matched with IOU and updated the tracking if the match of the predicted bounding box is successful. Otherwise, we create a new prediction bounding box, which is set to the uncertain and then performs the detection again.

It is seen that Kalman filtering is the key component of DeepSORT. However, the model accuracy determines the tracking accuracy, and the Kalman filter is a model-based algorithm. DeepSORT uses a classical tracking model based on the assumption of uniform speed, as shown in the following section.

### 3.3. Classical Tracking Model

In the two-dimensional plane, we assume the object is moving at a uniform speed. x[k] is defined as the object state at time *k*, including the object position $\left(p_{x}\left[k\right],p_{y}\left[k\right]\right)$, bounding box aspect ratio r[k], height h[k], and the object velocity $\left(v_{x}\left[k\right],v_{y}\left[k\right],v_{r}\left[k\right],v_{h}\left[k\right]\right)$. The details are expressed as follows:

(1) $$x\left[k\right]={\left(p_{x}\left[k\right],v_{x}\left[k\right],p_{y}\left[k\right],v_{y}\left[k\right],r\left[k\right],v_{r}\left[k\right],h\left[k\right],v_{h}\left[k\right]\right)}^{T}\in R^{8}$$

Take the *x*-axis object position $p_{x}$ as an example to explain the tracking model of the object. In addition, the *y*-axis object positions $p_{y}$, the bounding box aspect ratios *r*, and heights *h* follow the same model. According to the equation of uniform motion, the discrete form of the object position at k+1 can be recursively expressed by position $p_{x}\left[k\right]$ at time *k*, velocity $v_{x}\left[k\right]$ and system noise $w_{x}\left[k\right]$ as:

(2) $$p_{x}\left[k+1\right]=p_{x}\left[k\right]+v_{x}\left[k\right]\tau \left[k\right]+\frac{1}{2}\omega_{x}\left[k\right]\tau^{2}\left[k\right]$$

where *k* denotes the subscript of the sample, τ[k] indicates the sampling interval of the *k*th sample, and $\omega_{x}\left[k\right]$ is a Gaussian white noise with the mean 0, and the variance $\sigma_{\omega_{x}}^{2}$.

We denote the objective velocity vector $v_{x}\left[k+1\right]$ in discrete form at time k+1 as:

(3) $$v_{x}\left[k+1\right]=v_{x}\left[k\right]+w_{x}\left[k\right]\tau \left[k\right]$$

This is a classical object-tracking model. However, it hardly describes the acceleration motion of the object. In the actual tracking process, objects moving at a uniform speed are almost non-existent. Therefore, we propose a general tracking model with the variable acceleration motion in the next section.

## 4. The Proposed General Object Tracking Model

The complex motion of objects in videos and the occlusion problem motivate us to delve into the tracking model to achieve accurate and robust object tracking. In this section, we first devise a general object motion model with the classical tracking model and then build a Kalman filter tracking model to describe the complex situation.

### 4.1. General Motion Model

Due to the constant speed assumption of moving objects, it brings tracking delay and errors. In the actual moving process, there is no object moving at a constant speed. In addition, due to the occlusion problem caused by multi-object tracking, the constant speed assumption leads to the inaccuracy of object motion prediction, which further reduces the tracking performance. To better describe the acceleration state of the object, we build a general tracking model. Assuming that the object is in the accelerated motion, including the position $\left(p_{x}\left[k\right],p_{y}\left[k\right]\right)$, bounding box aspect ratio r[k] and height h[k], velocity $\left(v_{x}\left[k\right],v_{y}\left[k\right],v_{r}\left[k\right],v_{h}\left[k\right]\right)$, and the acceleration $\left(a_{x}\left[k\right],a_{y}\left[k\right],a_{r}\left[k\right],a_{h}\left[k\right]\right)$. Similarly, the tracking model of the object is described with the x-axis object position $p_{x}$ as an example. Similar to Equation (3), the acceleration $a_{x}\left[k\right]$ at k+1 th sampling period can be represented by the discrete tracking model as:

(4) $$a_{x}\left[k+1\right]=a_{x}\left[k\right]+\omega_{x}\left[k\right]\tau \left[k\right]$$

Similarly, we rewrite the velocity $v_{x}\left[k\right]$ as follows according to the variable acceleration motion mode of the object:

(5) $$v_{x}\left[k+1\right]=v_{x}\left[k\right]+a_{x}\left[k\right]\tau \left[k\right]+\frac{1}{2}\omega_{x}\left[k\right]\tau^{2}\left[k\right]$$

Therefore, a general model of object tracking is developed for the accelerated motion model as follows:

(6) $$x\left[k\right]=x\left[k-1\right]+v\left[k\right]\tau \left[k\right]+\frac{1}{2}a\left[k\right]\tau^{2}\left[k\right]+\frac{1}{6}\omega \left[k\right]\tau^{3}\left[k\right]$$

where $\omega_{x}\left[k\right]\tau \left[k\right]$ and $\frac{1}{2}\omega_{x}\left[k\right]\tau^{2}\left[k\right]$ are the system noise of velocity and acceleration, respectively. In addition, $\frac{1}{6}\omega \left[k\right]\tau^{3}\left[k\right]$ denotes the system disturbance of the object since the double integration of acceleration.

Note that our discrete tracking model is general. For relatively stable objects, the model corresponds to the classical tracking model if the acceleration is ignored. Our model is still reasonable if the acceleration is a constant rather than 0 or some other value that varies with time.

### 4.2. Multi-Object Tracking System

Based on the general model designed in the paper, we define the tracking system of the object as follows:

(7) $$x\left[k\right]={\left(p_{x}\left[k\right],v_{x}\left[k\right],a_{x}\left[k\right],p_{y}\left[k\right],v_{y}\left[k\right],a_{y}\left[k\right],r\left[k\right],v_{r}\left[k\right],a_{r}\left[k\right],h\left[k\right],v_{h}\left[k\right],a_{h}\left[k\right]\right)}^{T}\in R^{12}$$

(8) x[k+1]=Fx[k]+Gw[k]

where x[k+1] means object state at time k+1, *F* is transfer matrix applied to the previous state x[k], *G* represents the noise driver matrix, and $w\left[k\right]={\left(\omega_{x}\left[k\right],\omega_{y}\left[k\right],\omega_{r}\left[k\right],\omega_{h}\left[k\right]\right)}^{T}$ means the system noise vector at time *k* with covariance matrix $Q=\mathrm{diag}(\sigma_{x}^{2},\sigma_{y}^{2},\sigma_{r}^{2},\sigma_{h}^{2})$.

(9) $$F=\mathrm{diag}(F^{\prime },F^{\prime },F^{\prime },F^{\prime })$$

(10) $$F^{\prime }=\left[\begin{array}{ccc} 1 & t & \frac{t^{2}}{2}\\ 0 & 1 & t\\ 0 & 0 & 1 \end{array}\right]$$

(11) $$G=\mathrm{diag}(G^{\prime },G^{\prime },G^{\prime },G^{\prime })$$

(12) $$G^{\prime }={\left[\begin{array}{ccc} \frac{t^{3}}{6} & \frac{t^{2}}{2} & t \end{array}\right]}^{T}$$

The bounding box detected by YOLOv5 at time *k* is utilized as the observation z[k], including the object position $\left(p_{x}\left[k\right],p_{y}\left[k\right]\right)$, aspect ratio r[k], and height h[k], and $u\left[k\right]={\left(u_{x}\left[k\right],u_{y}\left[k\right],u_{r}\left[k\right],u_{h}\left[k\right]\right)}^{T}$ means the observation noise at time *k* with a mean value of 0 and covariance matrix $R=diag(\sigma_{ux}^{2},\sigma_{uy}^{2},\sigma_{ur}^{2},\sigma_{uh}^{2})$. Therefore, the measurement can be obtained as follows:

(13) z[k]=Hx[k]+u[k]

(14) $$H=\mathrm{diag}(H^{\prime },H^{\prime },H^{\prime },H^{\prime })$$

(15) $$H^{\prime }=\left[1,0,0\right]$$

Thus, we obtain the state system for object tracking. When the acceleration is not 0, the object tracking can be considered an accelerated motion model. According to the above general tracking model, we introduce the improved DeepSORT algorithm based on the unscented Kalman filter in the next section.

## 5. The Improved Multi-Object Tracking Algorithm

**Figure 2:**
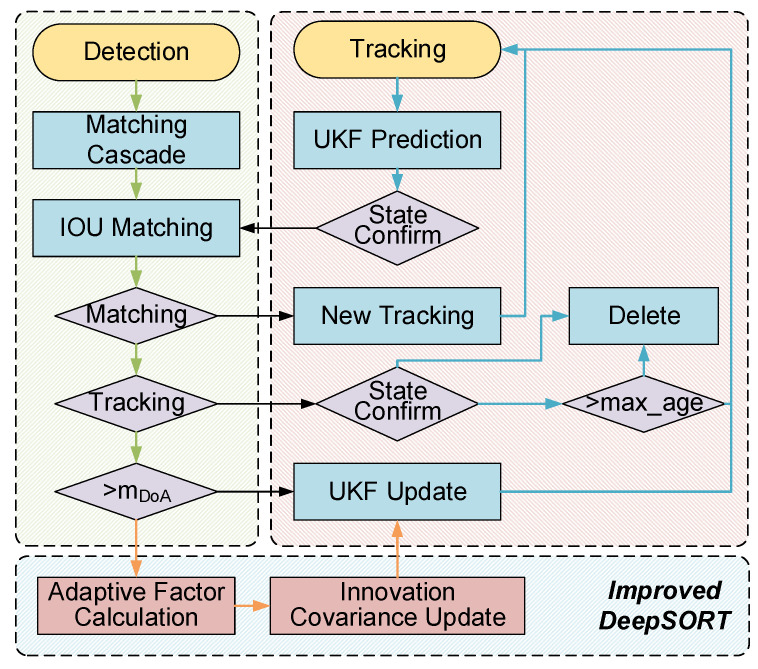
Improved DeepSORT algorithm framework.

Considering the accelerated motion model, the degree of nonlinearity of the system is exacerbated by the uncertainty caused by occlusion, noise, etc. The performance of the classical Kalman filter on nonlinear motion is not satisfactory. Thus, we design an improved DeepSORT algorithm based on the unscented Kalman filter for multi-object tracking. In addition, the results of the detection algorithm YOLOv5 are utilized as the observations, which are severely disturbed by random observation noise during the multi-object tracking process. Therefore, we propose an adaptive unscented Kalman filter by adjustment of the observation noise covariance matrix to enhance the tracking robustness and accuracy. The improved DeepSORT algorithm framework is shown in Figure 2.

### 5.1. Unscented Kalman Filter-Based Object Tracking Algorithm

Considering the multi-object tracking system (8) and (13), we select the following 2L+1 Sigma points at time *k* by the unscented transformation:

(16) $$X_{i}\left[k\right]=\left\{\begin{array}{c} \hat{x}\left[k\right]+\sqrt{\left(L+\lambda )P[k\right]},i=1,\ldots ,L\\ \hat{x}\left[k\right]-\sqrt{\left(L+\lambda )P[k\right]},i=L+1,\ldots ,2L\\ \hat{x}\left[k\right],i=0 \end{array}\right.$$

where $\hat{x}\left[k\right]$ and P[k] mean the state of multi-object tracking and the error covariance matrix at time *k*, respectively. In addition, *L* represents the dimension of the state vector, and $\lambda =\alpha^{2}\left(L+\kappa \right)-L$ is the distance parameter that controls the distribution of Sigma points. α and κ are scale parameters. The generated Sigma points are transformed by the state transfer matrix as follows:

(17) $$X_{i}\left[k+1|k\right]=FX_{i}\left[k\right],i=0,\ldots ,2L$$

Thus, we can obtain a priori estimation of the multi-object tracking state by time prediction and its corresponding error covariance matrix, which are denoted as $\hat{x}\left[k+1\;|\;k\right]$ and P[k+1∣k], respectively:

(18) $${\hat{x}}_{k+1|k}=\sum_{i=0}^{2L}w_{i}^{m}X_{i}\left[k+1|k\right]$$

(19) $$\begin{array}{c} P\left[k+1|k\right]=\sum_{i=0}^{2L}w_{i}^{c}\left(X_{i}\left[k+1|k\right]-\hat{x}\left[k+1|k\right]\right)\\ \begin{array}{ccc} & & \;{\left(X_{i}\left[k+1|k\right]-\hat{x}\left[k+1|k\right]\right)}^{T}+Q \end{array} \end{array}$$

where weight $w_{i}^{m}$ and $w_{i}^{c}$ are defined as follow:

(20) $$w_{0}^{m}=\frac{\lambda }{L+\lambda },\;w_{0}^{c}=\frac{\lambda }{L+\lambda }+\left(1-\alpha^{2}+\beta \right)$$

(21) $$w_{i}^{m}=w_{i}^{c}=\frac{\lambda }{2(L+\lambda )}$$

where β means the state distribution parameter, and the generated Sigma points are transformed by the measurement function as follows:

(22) $$Z_{i}\left[k+1|k\right]=HX_{i}\left[k+1|k\right],i=0,1,\ldots ,2L$$

Then, the mean, mutual covariance matrix, and error covariance matrix of transformed Sigma points are obtained as:

(23) $$\hat{z}\left[k+1|k\right]=\sum_{i=0}^{2L}w_{i}^{m}Z_{i}\left[k+1|k\right]$$

(24) $$\begin{array}{c} P_{zz}\left[k+1|k\right]=\sum_{i=0}^{2L}w_{i}^{c}\left(Z_{i}\left[k+1|k\right]-\hat{z}\left[k+1|k\right]\right)\\ \;{\left(Z_{i}\left[k+1|k\right]-\hat{z}\left[k+1|k\right]\right)}^{T}+R \end{array}$$

(25) $$\begin{array}{c} P_{xz}\left[k+1|k\right]=\sum_{i=0}^{2L}w_{i}^{c}\left(X_{i}\left[k+1|k\right]-\hat{x}\left[k+1|k\right]\right)\\ \;{\left(Z_{i}\left[k+1|k\right]-\hat{z}\left[k+1|k\right]\right)}^{T} \end{array}$$

Therefore, we can obtain the posterior estimation and the corresponding error covariance matrix after the observation update as follows:

(26) $$K\left[k\right]=P_{xz}\left[k+1|k\right]{\left(P_{zz}\left[k+1|k\right]\right)}^{-1}$$

(27) $$\begin{array}{c} \hat{x}\left[k+1\right]=\hat{x}\left[k+1|k\right]+K\left[k\right]\left(z\left[k+1\right]-\hat{z}\left[k+1|k\right]\right) \end{array}$$

(28) $$P\left[k+1\right]=P\left[k+1|k\right]-K\left[k\right]P_{zz}\left[k+1|k\right]K^{T}\left[k\right]$$

where K[k] represents the Kalman filter gain matrix.

The object-tracking algorithms of DeepSORT based on the UKF are suitable for handling nonlinear visual information and can provide reliable object-tracking estimates. However, mutual occlusion and interference in the process of multi-object tracking, as well as complex spatial relationships and the randomness of the number of objects, can bring about tracking uncertainty, resulting in unpredictable random interference noise. In addition, accurate object detection determines the performance of tracking. Due to the above factors, the inaccuracy of object detection based on YOLOv5 leads to an increase in observation error and inaccuracy of observation, which seriously reduces the tracking performance. Therefore, the noise matrix has to be corrected for accurate multi-object tracking. In the next section, we adjust the innovation covariance matrix by introducing an adaptive factor.

### 5.2. Improved Unscented Kalman Filter Algorithm

Due to the inaccuracy of the object detection results as observations, we have to detect and correct outliers. We introduced the concept of DoA [52] as an evaluation metric for the observation noise level. The innovation covariance matrix, according to the definition, can be expressed as follows:

(29) $$\begin{array}{cc} P_{f}\left[k+1\right] & =E\left(e\left[k+1\right]e^{T}\left[k+1\right]\right)\\ \; & =HP\left[k+1|k\right]H^{T}+R \end{array}$$

where $e\left[k+1\right]=z\left[k+1\right]-\hat{z}\left[k+1|k\right]$ indicates innovation sequence.

In addition, the innovation covariance matrix is:

(30) $$P_{e}\left[k+1\right]=P_{zz}\left[k+1|k\right]$$

To simplify the calculation of DoA, we take the diagonal elements of the innovation covariance matrix and represent them as:

(31) $$D_{f}\left[k+1\right]=\mathrm{diag}\left(P_{f}\left[k+1\right]\right)$$

(32) $$D_{e}\left[k+1\right]=\mathrm{diag}\left(P_{e}\left[k+1\right]\right)$$

Thus, DoA can be described as [52]:

(33) $$\mathrm{DoA}\left[k+1\right]=\mathrm{trace}\left(\frac{D_{f}\left[k+1\right]}{d\cdot D_{e}\left[k+1\right]\cdot \left(2-\alpha^{2}-\beta \right)}\right)$$

where *d* means the dimensionality of the observation vector, α and β are the system parameters. The mathematical expectation of DoA is $m_{DoA}=E\left(DoA\left[k+1\right]\right)=1$.

According to the definition of DoA, we introduce an adaptive factor to adjust the observation noise covariance:

(34) $$\lambda \left[k\right]=\left\{\begin{array}{c} 1,DoA\left[k\right]\leq m_{DoA}\\ \lambda^{*}\left[k\right],DoA\left[k\right]>m_{DoA} \end{array}\right.$$

When $DoA\left[k+1\right]>m_{DoA}$, the corrected innovation covariance matrix is:

(35) $$P_{zz}\left[k+1|k\right]=P_{zz}\left[k+1|k\right]+\left(\lambda^{*}\left[k+1\right]-1\right)R$$

Considering that $P_{zz}\left[k+1|k\right]$ is a function of $\lambda^{*}\left[k+1\right]$, we minimize the following equation to obtain $\lambda^{*}\left[k+1\right]$:

(36) $$\min\left\{J\left(\lambda^{*}\left[k+1\right]\right)={∥P_{f}\left[k+1\right]-P_{zz}\left(\lambda^{*}\left[k+1\right]\right)∥}^{2}\right\}$$

where ${∥M∥}^{2}$ is expressed as a parametrization of *M*, ${∥M∥}^{2}=\mathrm{trace}\left(MM^{T}\right)$.

For convenience, we let:

(37) $$\begin{array}{c} A=\sum_{i=0}^{2L}w_{i}^{c}\left(Z_{i}\left[k+1|k\right]-\hat{z}\left[k+1|k\right]\right)\\ {\left(Z_{i}\left[k+1|k\right]-\hat{z}\left[k+1|k\right]\right)}^{T} \end{array}$$

Thus, $P_{zz}\left(\lambda^{*}\left[k+1\right]\right)=A+\left(\lambda^{*}\left[k+1\right]-1\right)R$, and we have the following expression:

(38) $$\lambda^{*}\left[k+1\right]=\frac{\mathrm{trace}\left\{\left(P_{f}\left[k+1\right]-A\right)R^{T}\right\}}{\mathrm{trace}\left\{RR^{T}\right\}}$$

The proof procedure of the above equation is essentially the same as that of [53]. Finally, an improved DeepSORT multi-object tracking algorithm with the adaptive unscented Kalman filter is implemented with YOLOv5.

## 6. Experimental Evaluation

We carry out experiments on the MOT16 dataset for multi-object tracking to verify the feasibility of the improved DeepSORT algorithm. The hardware configuration uses Intel Xeon Gold 5120 CPU processor and NVIDIA GTX 2080Ti. The software environment uses Ubuntu 20.04 OS, CUDA10.1, OpenCV4.1.2, and uses Pytorch as the deep learning framework.

### 6.1. MOT16 Dataset Evaluation

Many existing methods utilize the YOLO object detection method as input for object tracking in their works. However, the latest YOLOv5 is rarely utilized. Therefore, this paper adopts YOLOv5l as the detection input and utilizes the labels of MOT16 as the ground truth. We compare the performance of the proposed improved DeepSORT method with the original DeepSORT and the existing baseline algorithm [54] in this case.

**Figure 3:**
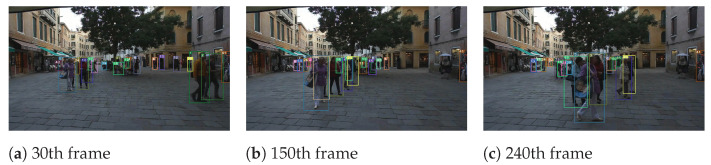
The tracking visualization of Mot16-02.

**Figure 4:**
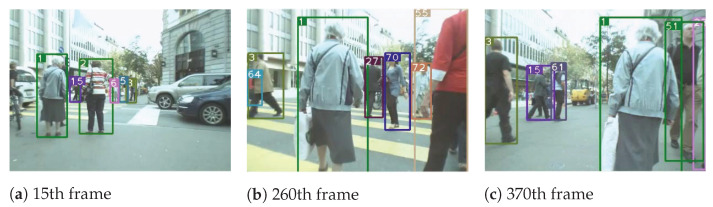
The tracking visualization of Mot16-05.

**Figure 5:**
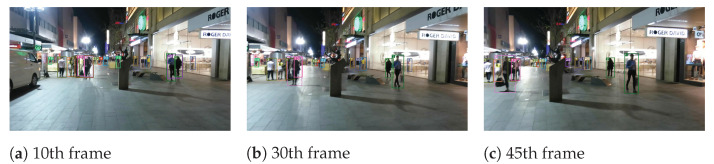
The tracking visualization of Mot16-10.

**Figure 6:**
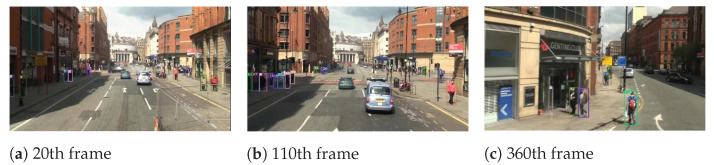
The tracking visualization of Mot16-13.

To reflect the multi-object tracking performance, object number 5 in the MOT15-02 sequence, object number 1 in the MOT15-05 sequence, object number 1 in the MOT15-10 sequence, and object number 10 in the MOT15-13 sequence are visualized. We can see from Figure 3, Figure 4, Figure 5 and Figure 6 that our algorithm in this paper continuously tracks object number 1 from frames 15, 260 to 370, and object number 10 from frames 20, 110 to 360, etc., showing a satisfactory tracking effect.

**Figure 7:**
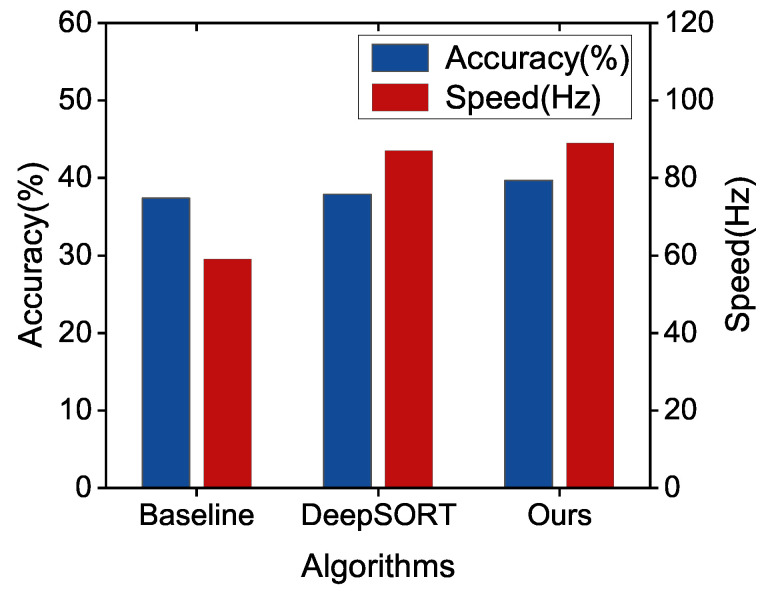
Performance Comparison between improved DeepSORT and DeepSORT with YOLOv5l.

The tracking results are further presented in Figure 7. We take multi-object tracking accuracy (MOTA) and running speed as evaluation indicators. We can see that the improved DeepSORT shows better performance in both speed and accuracy.

To better describe the effectiveness of our algorithm, we present the performance evaluation under various sequences on MOT16, and more evaluation indicators, as shown in Table 1. It presents that the improved DeepSORT algorithm achieves higher multi-object tracking accuracy (MOTA) scores and fewer false positives (FP) and false negatives (FN) than the DeepSORT algorithm in the MOT16 training sequence.

In addition, the switching times of object ID numbers (IDS) are also reduced. Another interesting finding is that the improved DeepSORT achieves better performance from dynamic cameras (MOT 16-05, MOT 16-10, MOT 16-11, and MOT 16-13). Due to the introduction of unscented Kalman filtering and the adaptive adjustment factors, the nonlinear error caused by dynamic cameras is reduced. The most important is that the improved scheme can improve not only the accuracy but also the multi-object tracking speed significantly. This is because the improved DeepSORT scheme builds a general tracking model, which can provide better bounding box prediction and shorten the processing and time of uncertain bounding boxes.

As we can see from Table 1, compared with the baseline algorithm, the speed and accuracy of our proposed algorithm improves by 33.71%and 6.15%. Note that ‘↑’ stands for rising and ‘↓’ stands for falling In addition, the improved DeepSORT scheme enhances the speed by 2.30% and the accuracy by 4.75% compared with the DeepSORT algorithm.

### 6.2. Tracking Performance Comparison under Different Detection Models

To investigate the impact of detection algorithms on tracking performance, the detection results from YOLOv5x, YOLOv5m, and YOLOv5s are utilized as inputs, respectively. The performance of the YOLOv5 under various models is presented in Table 2, where mAP represents the mean accuracy. We can see that the detection accuracy gradually degrades as the model size decreases. In addition, the detection speed gradually increases as the model size decreases.

**Figure 8:**
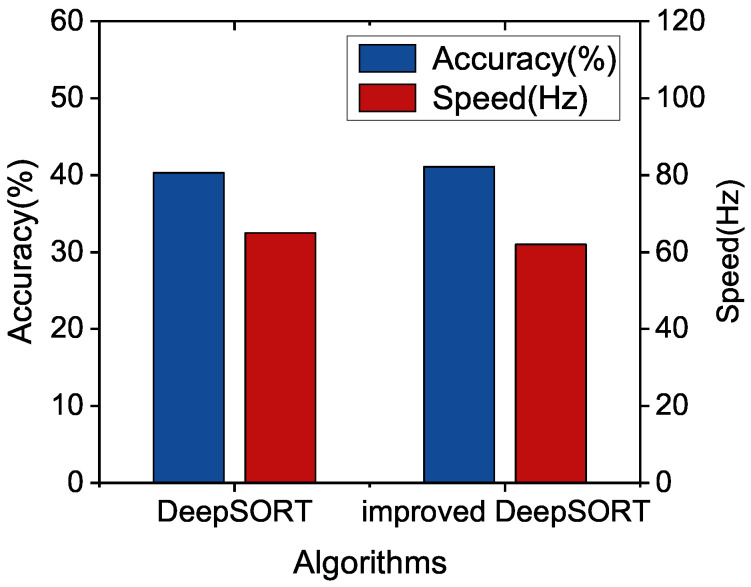
Performance Comparison between improved DeepSORT and DeepSORT with YOLOv5x.

**Figure 9:**
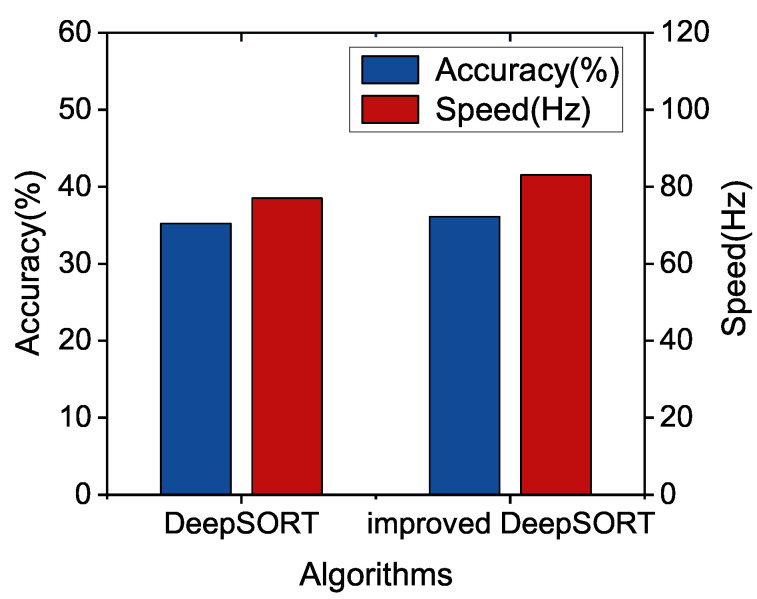
Performance Comparison between improved DeepSORT and DeepSORT with YOLOv5m.

**Figure 10:**
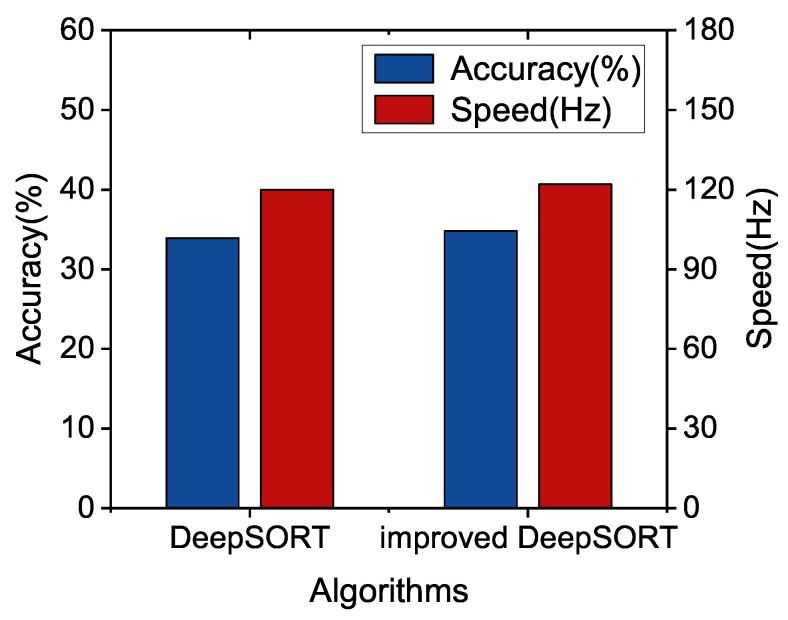
Performance Comparison between improved DeepSORT and DeepSORT with YOLOv5s.

Furthermore, we compare the tracking performance of our algorithm with the DeepSORT algorithm based on different YOLOv5 detection models in the camera video sequence MOT16. It is shown in Figure 8, the precision of improved DeepSORT with YOLOv5x is increased by 1.99%, but the speed is decreased by 4.62%. For too large models, The proposed algorithm may not improve the speed for the too large model significantly. In Figure 9, we can see that the speed and accuracy of improved DeepSORT with YOLOv5m are increased by 2.49% and 7.79%. Finally, it can be observed from Figure 10 that improved DeepSORT with the smallest YOLOv5s are increased by 2.65% and 1.67%, separately. We found that the detection model that is too small or too large may not enhance the tracking performance, and we have to select the appropriate detection model.

As can be seen from Table 3, we describe more detailed performance indicators and evaluation of multi-object tracking under different detection models. We can see that the proposed algorithm has been improved to varying degrees for different detection models. Both DeepSORT and improved DeepSORT improve performance with the improvement of the quality of test results. It has better accuracy under higher quality detection but low processing speed.

**Figure 11:**
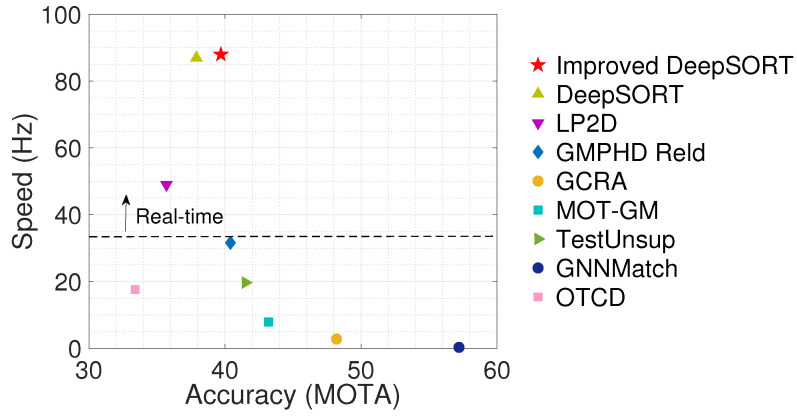
Performance Comparison between improved DeepSORT and existing tracking algorithms.

In addition, we compared the accuracy and speed of improved DeepSORT with several advanced methods, as shown in Figure 11. The results indicate that the improved DeepSORT method can obtain better accuracy results at a higher speed compared with the other tracking methods. Algorithms with higher accuracy than our method are far slower in speed and cannot reach real-time. In addition, the algorithm that is faster than the proposed algorithm is far less accurate. Our algorithm achieves a balance between accuracy and speed. Therefore, we can conclude that when the detection quality is appropriate, the algorithm proposed in this paper is more effective than DeepSORT.

## 7. Conclusions

This paper proposes an improved DeepSORT algorithm based on the unscented Kalman filter for multi-object tracking. First, a more realistic general object tracking model is developed. Then, an unscented Kalman filter-based object tracking algorithm is proposed, and an adaptive factor is introduced. Thus, the effect of nonlinear error, occlusion, and fast motion on the object tracking accuracy is reduced. Multi-object tracking is achieved at the algorithmic level rather than in terms of network models. The results indicate that the improved DeepSORT method has a lower computational cost and better tracking accuracy with 4.75% improvement in accuracy and 2.30% improvement in speed compared with DeepSORT. It can be better applied in practical scenarios.

The existing object tracking based on detection depends on the accuracy and speed of object detection. Our future works mainly focus on improving the performance of object tracking by improving the performance of object detection. In addition, we improve the data association algorithm for the occlusion between multiple objects in the tracking process to reduce the tracking error rate and the number of conversions between objects. 

## Figures and Tables

**Table 1:** The tracking results of MOT 16 sequence based on YOLOv5l.

Sequence	Method	MOTA↑	FP↓	FN↓	IDS↓	Hz↑
MOT16-02	Baseline	23.8	2992	14,982	86	46
	DeepSORT	22.9	2952	14,593	96	61
	improved DeepSORT	23.5	2976	14,613	83	59
MOT16-04	Baseline	33.5	4277	35,355	89	52
	DeepSORT	25.9	4677	37,059	91	81
	improved DeepSORT	26.4	4093	36,509	70	83
MOT16-05	Baseline	39.4	3313	4472	128	81
	DeepSORT	45.7	3146	4349	104	147
	improved DeepSORT	49.4	2920	4170	96	152
MOT16-09	Baseline	56.9	2633	3098	64	97
	DeepSORT	61.0	1927	2925	50	112
	improved DeepSORT	61.3	1419	2440	48	116
MOT16-10	Baseline	37.2	2820	8274	87	66
	DeepSORT	39.3	2937	7827	80	85
	improved DeepSORT	41.6	2672	7638	68	86
MOT16-11	Baseline	51.3	3270	5179	27	56
	DeepSORT	49.2	3893	5408	30	95
	improved DeepSORT	50.6	3081	4623	28	94
MOT16-13	Baseline	19.2	3240	9993	114	17
	DeepSORT	21.3	2659	9328	105	29
	improved DeepSORT	24.8	2145	8929	90	31
Total	Baseline	37.4	22,545	81,353	595	59
	DeepSORT	37.9	22,191	81,489	556	87
	improved DeepSORT	39.7	19,306	78,922	483	89

**Table 2:** The performance comparison of various detection models.

Detection Model	mAP	Hz	Model Size
YOLOv5x	49.6	145	89.0 M
YOLOv5l	47.2	201	40.2 M
YOLOv5m	44.5	294	21.8 M
YOLOv5s	37.0	416	7.5 M

**Table 3:** The tracking performance with various detection models.

Detection Input	Method	MOTA	FP	FN	IDS	Hz
YOLOv5x	DeepSORT	40.3	20,555	77,307	504	65
	improved DeepSORT	41.1	19,494	76,494	454	62
YOLOv5m	DeepSORT	35.2	22,902	77,936	590	77
	improved DeepSORT	36.1	19,991	75,243	528	83
YOLOv5s	DeepSORT	33.9	20,728	80,335	514	120
	improved DeepSORT	34.8	19,570	78,375	464	122

## Data Availability

Not applicable.
